# Forensic age prediction for saliva samples using methylation-sensitive high resolution melting: exploratory application for cigarette butts

**DOI:** 10.1038/s41598-017-10752-w

**Published:** 2017-09-05

**Authors:** Yuya Hamano, Sho Manabe, Chie Morimoto, Shuntaro Fujimoto, Keiji Tamaki

**Affiliations:** 1 Department of Forensic Medicine, Graduate School of Medicine, Kyoto University, Kyoto, Japan; 2 Forensic Science Laboratory, Kyoto Prefectural Police Headquarters, Kyoto, Japan

## Abstract

There is high demand for forensic age prediction in actual crime investigations. In this study, a novel age prediction model for saliva samples using methylation-sensitive high resolution melting (MS-HRM) was developed. The methylation profiles of *ELOVL2* and *EDARADD* showed high correlations with age and were used to predict age with support vector regression. *ELOVL2* was first reported as an age predictive marker for saliva samples. The prediction model showed high accuracy with a mean absolute deviation (MAD) from chronological age of 5.96 years among 197 training samples. The model was further validated with an additional 50 test samples (MAD = 6.25). In addition, the age prediction model was applied to saliva extracted from seven cigarette butts, as in an actual crime scene. The MAD (7.65 years) for these samples was slightly higher than that of intact saliva samples. A smoking habit or the ingredients of cigarettes themselves did not significantly affect the prediction model and could be ignored. MS-HRM provides a quick (2 hours) and cost-effective (95% decreased compared to that of DNA chips) method of analysis. Thus, this study may provide a novel strategy for predicting the age of a person of interest in actual crime scene investigations.

## Introduction

In forensic science, predicting the age of a victim or a suspect can trigger the quick solution of a crime. Nonetheless, forensic scientists have had few options for estimating the age of the person of interest in actual practice, such as examining bones morphologically^1^ or analysing the amino acid racemization of teeth^2^. These techniques are not versatile methods, as they limit sample sources. In addition, biological fluids, which are more commonly found at crime scenes, cannot be analysed with these morphological techniques. For this reason, forensic scientists have begun to apply knowledge of genetics to forensic cases, *e.g*. signal joint T-cell receptor excision circles (sjTREC)^3^, telomere length^4^, and somatic gene arrangements^5^. However, these genetic biomarkers exhibit relatively low accuracy or are severely influenced by the degradation of DNA collected from evidentiary materials found in actual crime scenes.

Epigenetics have recently come to play an important role in forensic age prediction. Cytosine methylation at CpG sites has been well investigated as a novel epigenetic marker of chronological age^6–17^. Hannum *et al*. built a predictive model for aging blood with 71 methylation markers selected from the Illumina Infinium HumanMethylation450 BeadChip, resulting in an error of 4.89 years^6^. Huang *et al*. also developed a predictive model for bloodstains using 5 CpG sites analysed by pyrosequencer with a mean absolute deviation (MAD) of 7.98 years^7^. Although these methods are novel, none are routinely applied for actual criminal investigations currently, likely because of their high cost and time requirements.

Traditional polymerase chain reaction (PCR), which is a universal and cost- and time-effective method, may be the key technique for the realization of forensic age prediction in actual crime investigations owing to its many advantages^18^. Recently, Mawlood *et al*. developed a useful age prediction method based on a qPCR systemme^19^. We have newly developed a novel age prediction model that involves the use of methylation-sensitive high resolution melting (MS-HRM) for blood samples^20^. Antunes *et al*. also described the application potential of MS-HRM for forensic use^21^. MS-HRM is a method that measures methylation profiles easily, quickly, and cost-effectively, where the PCR amplification of bisulphite-treated DNA is followed by melting analysis^22–24^. In bisulphite-treated DNA analysis, unmethylated cytosines are converted into uracils by bisulphite conversion while methylated cytosines are kept intact. Therefore, the methylation status of each cytosine is directly converted into the sequence, where it alters the thermodynamic stability of double-stranded DNA. Thus, a novel age prediction model that is suitable for actual crime investigations using MS-HRM has been developed.

However, in most of the studies performed previously^6–8, 10–17, 19, 20^, the research object has been limited to blood samples. DNA methylation profiles can differ depending on the cell type from which the DNA is derived^25–27^. Therefore, an age prediction model established from blood DNA may not be applicable for DNA derived from other biological fluids. To the best of our knowledge, only Bocklandt *et al*.^28^ and Horvath^9^ have investigated saliva samples, which are also commonly found at crime scenes. During the writing of this manuscript, Hong *et al*. also developed an age estimation model for saliva samples^29^. However, all of these methods suffer from the abovementioned difficulties in practical use.

Here, we report a practical age prediction method that involves analysing the methylation status of *ELOVL2* and *EDARADD* via MS-HRM of saliva samples. *ELOVL2* is newly reported to correlate with chronological age in saliva samples. In this study, 197 saliva samples were analysed to develop an age prediction model, and the model was further validated using 50 additional samples. The cost and time required for analysis were dramatically reduced with this method. In addition, saliva DNA was extracted from cigarette butts, and then age prediction was performed as in an actual crime scene for the first time ever. This HRM-based method has great potential for predicting age and is quite useful, especially when DNA data for the person of interest are not recorded in criminal databases.

## Results

### Identification of optimal age markers for saliva samples with MS-HRM

In previous work, we developed an age prediction model for blood samples by analysing methylation profiles of the promoter regions of *ELOVL2* and *FHL2* ^20^. The degrees of methylation for both these markers increased with chronological age in blood samples. Therefore, we first investigated whether these markers could be applied for the analysis of saliva samples with MS-HRM. The methylation profile of *ELOVL2* clearly correlated with the age of the saliva samples, while that of *FHL2* exhibited no correlation with chronological age in the preliminary test (Supplementary Fig. 1).

To identify another methylation marker for MS-HRM, the top five markers positively correlated with age (*KCNG3*, *NPTX2*, *GREM*, *VGF*, and *PDE4C*) and the top five negatively correlated with age (*ASPA*, *Bles03*, *EDARADD*, *TCEA2*, and *ELN*) were selected from the study of Bocklandt *et al*., in which Illumina HumanMethylation27 microarrays were used to analyse saliva samples^28^. Bisulphite PCR primers were newly designed for these 10 markers for HRM, though only *EDARADD* showed site-specific bisulphite PCR amplification due to the sequence simplicity of bisulphite-modified DNA (*i.e*. most cytosines are converted to uracils, which act as thymines in the PCR amplification process). Thus, *ELOVL2* and *EDARADD* were selected as age prediction candidate markers for use with MS-HRM of saliva samples. The sequences of the PCR primers used in this study are shown in Table 1.

**Table 1:** Sequences of PCR primers for *ELOVL2* and *EDARADD*.

Target marker		Sequence
ELOVL2	Fw	CGATTTGTAGGTTTAGT
	Rv	ACTACCAATCTAAACAA
EDARADD	Fw	AGAAGGTTTGATTTTGGTTAGAT
	Rv	CCTCTCCCCATCTATTTAAT

**Figure 1:**
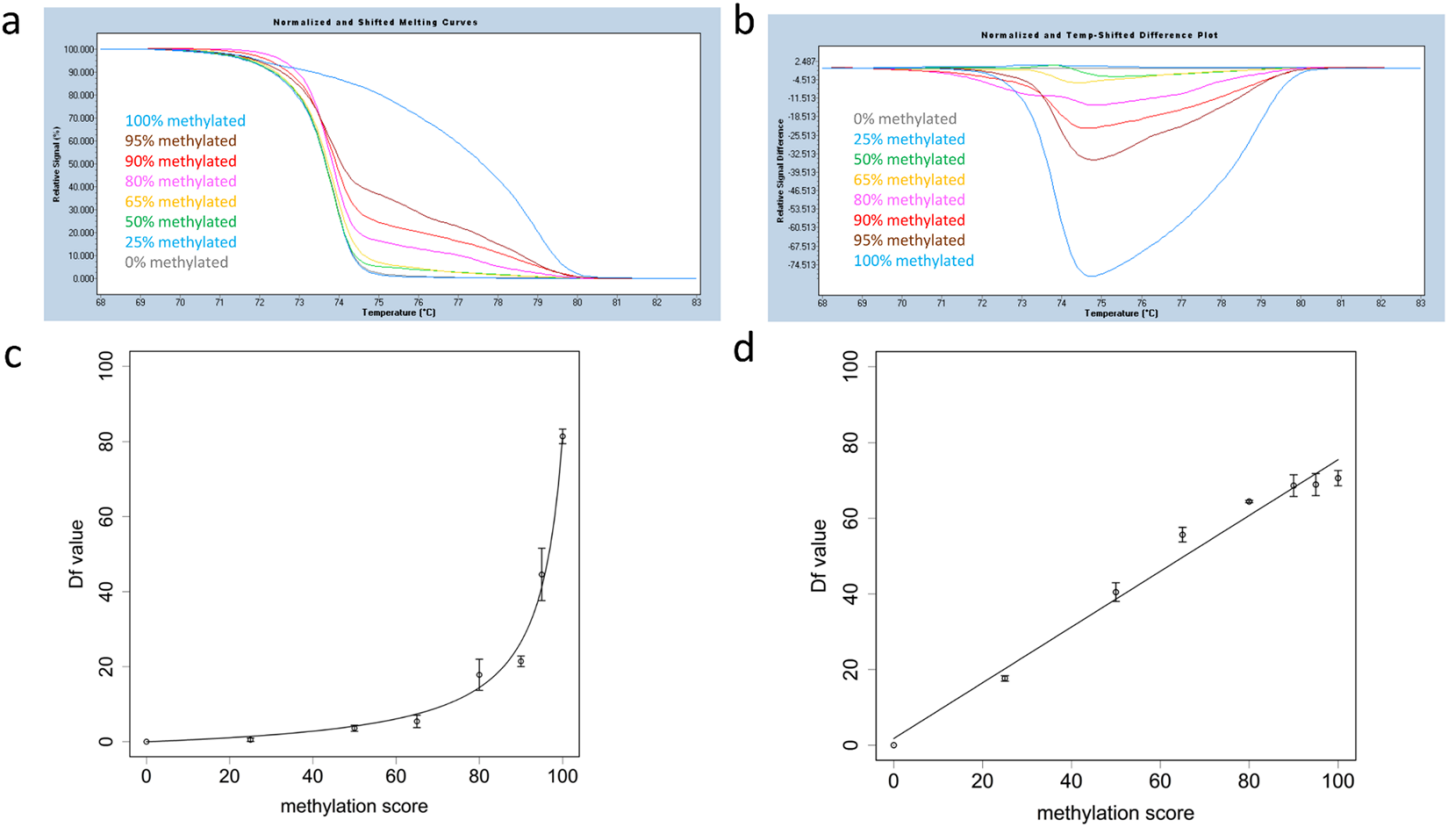
MS-HRM analysis of DNA methylation. (**a**) Schematic representation of MS-HRM. Normalized melting curve. Control DNA samples were mixed and adjusted to 0%, 25%, 50%, 65%, 80%, 90%, 95%, and 100% methylated. (**b**) Normalized difference plot of control DNA samples. Melting data of 0% methylated standard sample was set to baseline (grey). (**c**) Standard curve of *ELOVL2*. Error bars represent standard errors. (**d**) Standard line of *EDARADD*. Error bars represent standard errors.

PCR bias often occurs when amplifying bisulphite-treated DNA^30, 31^, since unmethylated DNA tends to be amplified more efficiently than methylated DNA. To analyse methylation profiles accurately, therefore, an interpolation line or curve must be obtained before measuring unknown methylated samples with MS-HRM. Thus, a standard line and curve were first established (Fig. 1). The promoter region of *ELOVL2* showed some PCR bias, as expected^20^. In contrast, *EDARADD* showed no PCR bias; thus, the standard line was linear. The maximum absolute relative signal difference values (Df values) obtained following HRM analysis of each sample were plotted, and a non-linear regression model was developed for *ELOVL2*, as depicted in Eq. (1):

1 $$\frac{0.054\times {M}_{1}}{100-{M}_{1}}=\frac{Df}{D{f}_{\max }-Df}$$

where M_1_ is the methylation score of *ELOVL2* and Df_max_ is the Df value of a 100% methylated control sample. For *EDARADD*, a simple linear regression model was developed, as depicted in eq. (2):

2 $${M}_{2}=1.765+0.737\times Df$$

where M_2_ is the methylation score of *EDARADD*. Thereafter, the methylation scores of *ELOVL2* and *EDARADD* were calculated by substituting the Df value into the corresponding regression model.

### Developing an age prediction model

**Figure 2:**
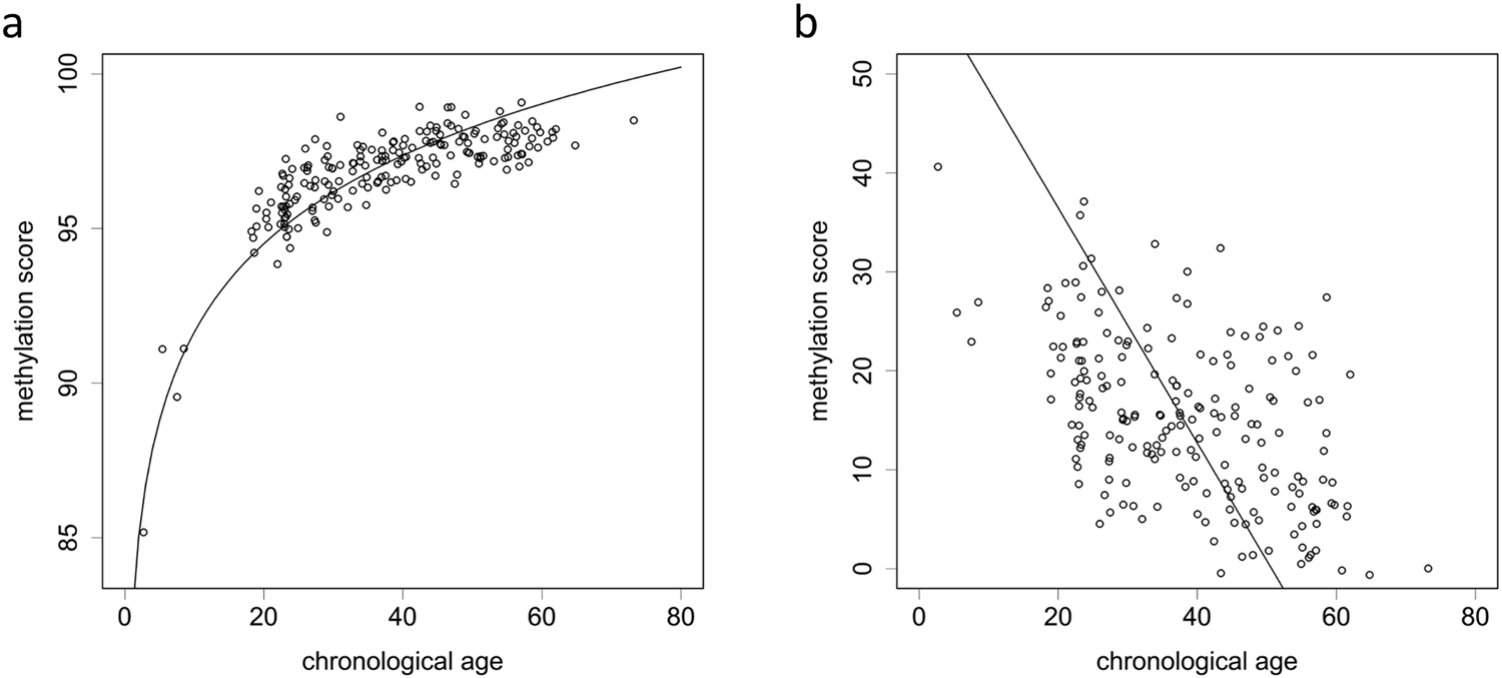
Relationship between age and methylation score for (**a**) *ELOVL2* and (**b**) *EDARADD*.

**Figure 3:**
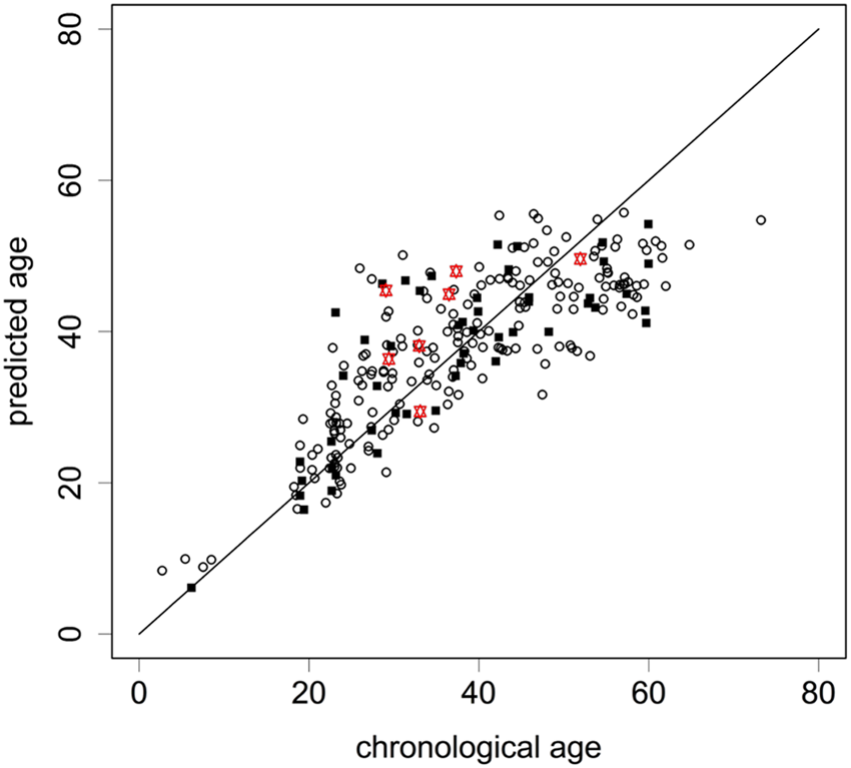
Correlation between predicted age and chronological age. In total, 197 training set samples plotted as white circles, 50 test set samples plotted as black squares, and seven cigarette butts plotted as red stars. The black line represents the y = x diagonal line.

Next, we analysed the methylation scores of *ELOVL2* and *EDARADD* in 197 saliva samples with MS-HRM (Fig. 2). Detailed information for the samples is shown in Table 2. *ELOVL2* was positively correlated with the logarithm of chronological age (Pearson’s correlation coefficient r = 0.868), while *EDARADD* showed a negative correlation (r = −0.519). The relationship between the methylation score and the chronological age fit the logarithmic curve well for *ELOVL2*. The methylation score of *EDARADD* showed a linear decrease with chronological age. No statistically significant difference was observed between male and female samples for either of the two markers when performing analysis of co-variance (ANCOVA) (Supplementary Fig. 2; p = 0.849 and 0.382 for *ELOVL2* and *EDARADD*, respectively). Subsequently, a final age prediction model was developed with support vector regression^16^ using information from both markers (Fig. 3). The MAD was 5.96 years for the training set (adjusted R^2^ = 0.69). Then, an additional independent set of 50 saliva samples was analysed to validate this model. The accuracy of the age prediction model was demonstrated with a MAD of 6.25 years for the test set (adjusted R^2^ = 0.60). However, the MAD was smaller for younger individuals than for seniors (Supplementary Table 1).

**Table 2:** Age and gender information for 197 training and 50 test samples used in this study.

	Training set		Test set	
	Male	Female	Male	Female
under 20	5	5	2	3
20–39	51	49	10	16
40–59	45	36	13	6
over 60	5	1

### Exploratory application

Until now, a few groups had developed age prediction models for saliva samples^9, 28, 29^ and buccal epithelial cells^9, 32, 33^. However, no researcher have yet examined the utility of these methods for the analysis of forensic trace evidence, such as cigarette butts. In this study, we extracted DNA from seven cigarette butts and performed age estimation as an exploratory application (Fig. 3). The applicability of our model to cigarette butts was thus demonstrated, although the MAD of 7.65 years was slightly higher than that of intact saliva.

**Figure 4:**
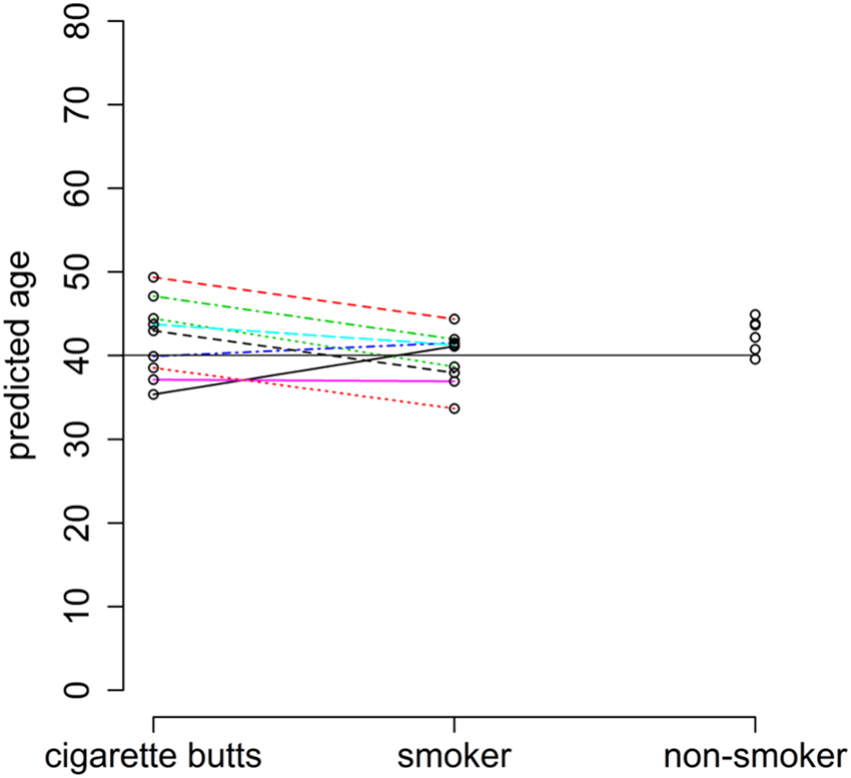
The results of age prediction for nine cigarette butts, nine smokers’ saliva samples, and seven non-smokers’ saliva samples. All sample donors were 40 years old. Cigarette butts and smokers’ saliva samples were collected from the same nine individuals (connected by straight lines).

Based on this, the effects of smoking habits and the ingredients in the cigarettes themselves were further examined. Tsaprouni *et al*. investigated the effect of a smoking habit on genome-wide DNA methylation and found some significant smoking-related markers^34^. The methylation statuses of 54 people (50 ± 1 years old) were retrieved from publicly available data sets (GSE50660), and the effect of a smoking habit was analysed for cg16867657 (*ELOVL2*) and cg09809672 (*EDARADD*) (Supplementary Fig. 3). No statistically significant differences were observed among non-, former, or current smokers according to analysis of variance (ANOVA; p = 0.075 and 0.332 for *ELOVL2* and *EDARADD*, respectively). Moreover, we collected nine cigarette butts and nine saliva samples from the same volunteers for use as smokers’ samples, as well as seven saliva samples from non-smokers. All of the sample donors were 40 years old. For these 25 samples (nine cigarette butts, nine smokers’ saliva samples, and seven non-smokers’ saliva samples), we analysed the methylation scores of *ELOVL2* and *EDARADD* with MS-HRM (Supplementary Fig. 4). No statistically significant differences in methylation scores were observed among cigarette butts, smokers’ saliva, or non-smokers’ saliva for *EDARADD* (ANOVA; p = 0.072). For *ELOVL2*, a statistically significant difference was observed (p = 0.012), but the difference was very slight. Subsequently, age predictions were successfully performed on these samples, resulting in MADs of 4.07, 2.56, and 2.79 years for cigarette butts, smokers’ saliva, and non-smokers’ saliva, respectively (Fig. 4). No statistically significant difference in prediction was observed among these categories according to ANOVA (p = 0.22). This demonstrates that the effect of a smoking habit and the contents of cigarettes themselves can be ignored when performing age prediction using the method developed in this study.

## Discussion

Age prediction has long been one of the most practically important goals for forensic scientists. Recently, novel age estimation models were developed by analysing the methylation degrees of some CpG markers for blood samples^6–8, 10–17^. However, none of these methods has been applied in actual crime investigations due to the high cost and extended length of time required for analysing DNA chips or pyrosequencing. In addition, only blood samples have been well investigated; thus, other forensically relevant body fluids—such as saliva—have been less discussed. The current study represents an age prediction model for saliva samples using MS-HRM, and it may solve the abovementioned problems of age prediction analysis.

MS-HRM is a real-time PCR-based technique that measures the integrated methylation statuses of multiple CpG sites in a single assay that is quick (2 hours) and cost-effective (approximately £3 for age prediction based on two markers). According to Mawlood *et al*., 35 hours are essential for pyrosequencing and next generation sequencing (NGS)^19^, which cost £75 and £90, respectively. Therefore, many other groups have begun to use MS-HRM for various aspects of forensic research, such as differentiating monozygotic twins^35^, identifying body fluids^21^, and discriminating between tigers^36^. Notably, Migheli *et al*. showed that MS-HRM gave estimates of *APC* and *CDKN2A* gene methylation that were similar to those obtained by pyrosequencing^37^. Amornpisutt *et al*. also referred to the presence of a significant agreement between MS-HRM and pyrosequencing^38^. However, MS-HRM has some disadvantages. The biggest may be that individual methylation rates cannot be measured by MS-HRM. For 427 blood samples, Zbieć-Piekarska *et al*. investigated the methylation rates of seven CpG sites in *ELOVL2* with pyrosequencing (from C1 to C7 in their study)^12^, which are also included in our analysing region with MS-HRM. The MAD of their model was 5.03 and 5.75 years for 303 training set and 124 test set, respectively. To evaluate the ability of MS-HRM in age prediction, another model was generated by performing support vector regression using the methylation score of *ELOVL2* only (the methylation score of *EDARADD* was not used). The MAD of this model (6.59 and 6.83 years for training set and test set, respectively) was a little higher than that of Zbieć-Piekarska’s model (Supplementary Table 2). It is important to note that there is the difference in body fluids; they investigated blood samples, but we analysed saliva samples.

In the study of Zbieć-Piekarska *et al*., the methylation rates of all seven CpG sites showed nearly the same correlation with chronological age (r = 0.798–0.913)^12^. Likewise, Garagnani *et al*. indicated that the methylation rates of CpG sites neighbouring an age-related CpG site were also associated with chronological age in *ELOVL2* ^8^. Moreover, Day *et al*. investigated the effect of age-related CpG sites to methylation on neighbouring CpG sites in detail^39^. In his research, age-related CpG sites that were proximal to the same gene region showed a ~91% overlap in association with age. These findings are consistent with our results that a certain level of accurate age prediction can be performed with MS-HRM. As mentioned previously, MS-HRM has its advantages in time and cost required for analysis. While less information is obtained with MS-HRM as compared to other techniques measuring individual CpG methylation rates; however, our model has a potential to provide scientists with another option to predict a subject’s age in an actual crime investigation and maybe useful to screen samples.

Another disadvantage may be the issue of PCR bias. In this study, the interpolation curve for *ELOVL2* showed non-linearity, indicating the presence of PCR bias, while *EDARADD* exhibited little PCR bias. Warnecke *et al*. proposed that the presence of PCR bias depends on the sequence of the bisulphite-treated DNA^30^. Thus, an interpolation curve must be obtained for each marker before analysing the methylation profile with MS-HRM, even when adapting the strategy for reversing PCR bias^31^.

The prediction accuracy of our model (MAD = 6.25 years) was a little lower than that of Bocklandt *et al*. (MAD = 5.2 years)^28^. As for blood samples, increasing the number of target sites tends to improve the age prediction accuracy. For example, Weidner *et al*. developed a prediction model with three CpG markers (MAD = 5.4 years), while a more accurate model required 102 markers (MAD = 3.34 years)^10^. Park *et al*. investigated the relationship between the age prediction accuracy and the number of target sites and suggested that the most preferable number of target sites might be three for practical reasons^40^. In this study, two markers were used to predict age; however, additional markers may improve the prediction accuracy. We initially selected 10 candidate CpG sites for age estimation using data from Illumina HumanMethylation27, which assesses 27,578 CpG sites. HumanMethylation450, which assesses > 450,000 CpG sites, may result in better candidate markers for enhancing prediction accuracy. Thus, further studies may be required to incorporate at least one more marker to establish a useful model for practical application.

The MAD was smaller for younger individuals than for seniors, which is consistent with the results of a study by Branicki^13^. In our study, the speed of methylation change of *ELOVL2* was significantly higher in youth. Thus, the prediction is more precise in young people. Addition of another CpG site that undergoes a change its methylation profile in older individuals will improve the accuracy of the predictive model in the senior segment of the population. *ELOVL2* is a promising age marker for blood samples^8, 12^, but methylation profiles for many CpG markers can change dramatically depending on cell type^41^. This is the first report to demonstrate the utility of *ELOVL2* in the determination of age using saliva samples. In our study, *EDARADD* (r = −0.519) showed a modest correlation coefficient compared to that of *ELOVL2* (r = 0.868). Huang *et al*. developed an age prediction model^7^ with four CpG markers ranging in absolute correlation coefficient (|r|) from 0.409 to 0.857. Higher marker correlations will also improve the age prediction model.

Individual lifestyles can cause changes in DNA methylation. The effect of a smoking habit on DNA methylation profiles has been particularly well investigated^34, 42^. According to previous studies, some loci (*AHRR*, *F2RL3*, etc.) showed significant differences in methylation between smokers and non-smokers. To the best of our knowledge, none of these smoking-associated markers were also identified as age-predictive markers. *ELOVL2* and *EDARADD* showed almost no relationship with smoking habit in this study; however further study might be required due to the small sample size of this study. Notably, the smoking habit did not significantly affect the accuracy of age prediction in our study. Thus, we conclude that when performing age prediction with saliva samples extracted from cigarette butts, any effects of a smoking habit or of the ingredients of cigarettes themselves can be ignored. Age prediction with nine cigarette butts from 40-year-old donors resulted in accurate predictions (MAD = 4.07 years), though the MAD of seven cigarette butts from volunteers ranging in age from 29 to 51 years was higher (MAD = 7.65 years). This difference may be attributed to the small sample size. In total, the MAD was 5.64 for 16 cigarette butts analysed in this study, although further research is necessary to support these findings. Saliva consists mainly of leucocytes and epithelial cells^43^. According to Weidner *et al*.^33^, a smaller MAD may be achieved by adding cell type markers to the prediction model.

In conclusion, a novel age prediction model for saliva samples using MS-HRM was developed in this study. There are three major points of caution before applying this method to actual forensic investigations. First, interpolation curves must be established for each instrument or reaction reagent, as the methylation score is affected by these conditions. Second, body fluid identification must be performed prior to age prediction. It is not appropriate to apply an age prediction model developed for saliva samples to blood or mixed samples. Third, since forensic samples are left in various conditions, the effect of prolonged storage and sample preservation methods must be investigated before applying this model to practice. When these requirements are fulfilled, the analysis of the methylation profiles of saliva samples with MS-HRM offers great potential for predicting age in actual crime scene investigations.

## Methods

### Ethic statement

All samples in this study were collected with permission for research use from the ethical committee of the Graduate School of Medicine of Kyoto University with approval number G1036. All experiments of this study were carried out in accordance with the Japanese ethical guidelines for human genome/gene analysis research, Ministry of Health, Labour and Welfare of Japan.

### Sample collection, DNA extraction, and bisulphite conversion

Saliva samples from 263 healthy donors ranging in age from 1 to 73 years were collected using plastic tubes. Cigarette butts were collected from 16 volunteers. All samples were immediately stored in a −30 °C freezer until use. All donors or their parents signed written consent forms including specific consent to publish the images in an online open-access publication prior to donation. Ethical approval was received from the ethical committee of the Graduate School of Medicine of Kyoto University. We obtained participants’ informed consent for all samples collected. For these samples, DNA was extracted and bisulphite-modified according to our previously published methods^20^.

### High resolution melting

PCR primers were designed with either BiSearch^44, 45^ or manually. For *ELOVL2*, the amplicon is 91 bp long and includes 10 CpG markers between primer binding sites (chr6: 11,044,611–11,044,701; UCSC Genome Browser GRCh38). For *EDARADD*, the amplicon is 139 bp long and includes four CpG sites (chr1: 236,394,341–236,394,480). PCR amplification was carried out with a Roche LightCycler 480 Instrument II (Roche Diagnostics GmbH, Mannheim, Germany) equipped with the Gene Scanning Software (version 1.5.1.62 SP2) in a 25 μL total volume containing 1 × EpiTect HRM PCR Master Mix, 250 nM of each primer, and 10 ng of bisulphite-modified template. When HRM analysis was performed, we set the pre-melt temperature region to 68–69 °C and the post-melt temperature region to 82–83 °C for *ELOVL2*. For *EDARADD*, these were set to 65–66 °C and 80–81 °C, respectively. In total, 263 saliva samples (197 in the training set, 50 in the test set, and 16 to examine the effect of smoking) were analysed using HRM in duplicate. Other variables were set appropriately according to our previous methods^20^.

### Calculating methylation scores

Fully methylated control DNA and fully unmethylated DNA were purchased from Qiagen (Hilden, Germany) and mixed in appropriate ratios to make 0%, 25%, 50%, 65%, 80%, 90%, 95%, and 100% methylated control DNA. The Df value of each sample obtained by HRM was plotted, and a non-linear regression model was developed for *ELOVL2* with R (version 3.2.2)^46^ using the “nls” command. For *EDARADD*, a simple linear regression model was developed with R using the “lm” command. HRM measurements were performed in triplicate to obtain the interpolation curve or line. We newly defined the methylation score, since HRM provides the overall methylation profile of PCR-amplified products rather than the methylation rates of the individual CpG markers. The methylation rates of all CpG markers present in the region of interest were integrated to determine the value of the methylation score and analysed with one pair of PCR primers in one measurement.

### Developing an age prediction model

First, to predict age, a non-linear regression model for *ELOVL2* was built from 197 saliva samples with R using the “nls” command. For *EDARADD*, a linear regression model was built using the “lm” command. Secondly, ANCOVAs were performed with IBM SPSS Statistics 20 to determine whether gender affected the regression models (p < 0.05 was considered statistically significant). Finally, a support vector regression model was built using the “e1071” package^47^. Support vector regression parameters were optimized with “tune.svm” command and set as “Cost = 1.1, gamma = 0.1”. The final model was further validated using an additional set of 50 test samples.

### Assessing the impact of smoking

The methylation profiles of 54 people ranging in age from 49 to 51 years were retrieved from a publicly available dataset (GSE50660)^34^. They were categorized into three groups by their smoking habits (non-smokers, former smokers, and current smokers) according to Tsaprouni^34^. ANOVA was performed for those data with R using the “anova” and “aov” commands (p < 0.05 was considered statistically significant). In addition, to evaluate if a smoking habit or the ingredients of the cigarettes themselves affected the methylation score, we collected nine cigarette butts and nine saliva samples from the same volunteers for use as smokers’ samples, as well as seven saliva samples from non-smokers. All sample donors were 40 years old. ANOVAs were performed on the methylation scores and the predicted ages of these samples with R using the “anova” and “aov” commands with default settings.

### Availability of data and material

The datasets generated during and/or analysed during the current study are not publicly available due to protecting participant confidentiality but are available from the corresponding author on reasonable request.

## Electronic supplementary material

Supplementary Information
