# Supplementary material for: Forensic age prediction for saliva samples using methylation-sensitive high resolution melting: exploratory application for cigarette butts
Source: Sci Rep. 2017 Sep 5;7:10444. doi: 10.1038/s41598-017-10752-w (PMC5585169; doi:10.1038/s41598-017-10752-w)
Supplement: Supplementary file 1 — Supplementary Information [file 41598_2017_10752_MOESM1_ESM.doc]

**Supplementary Information**

**Forensic age prediction for saliva samples using methylation-sensitive high resolution melting: exploratory application for cigarette butts**

Yuya Hamano^1, 2^, Sho Manabe^1^, Chie Morimoto^1^, Shuntaro Fujimoto^1^, Keiji Tamaki^1^

^1^Department of Forensic Medicine, Graduate School of Medicine, Kyoto University, Japan. ^2^Forensic Science Laboratory, Kyoto Prefectural Police Headquarters, Japan. Correspondence and requests for materials should be addressed to K. T. (email: ktamaki@fp.med.kyoto-u.ac.jp)


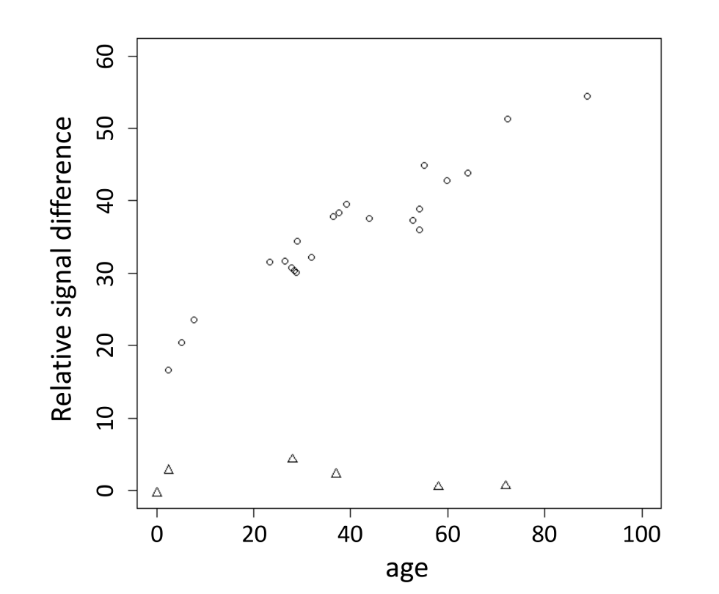


Supplementary Figure 1. Distribution of differential value of FHL2 and chronological age (circle; blood samples in our previous work^1^, triangle; saliva samples).


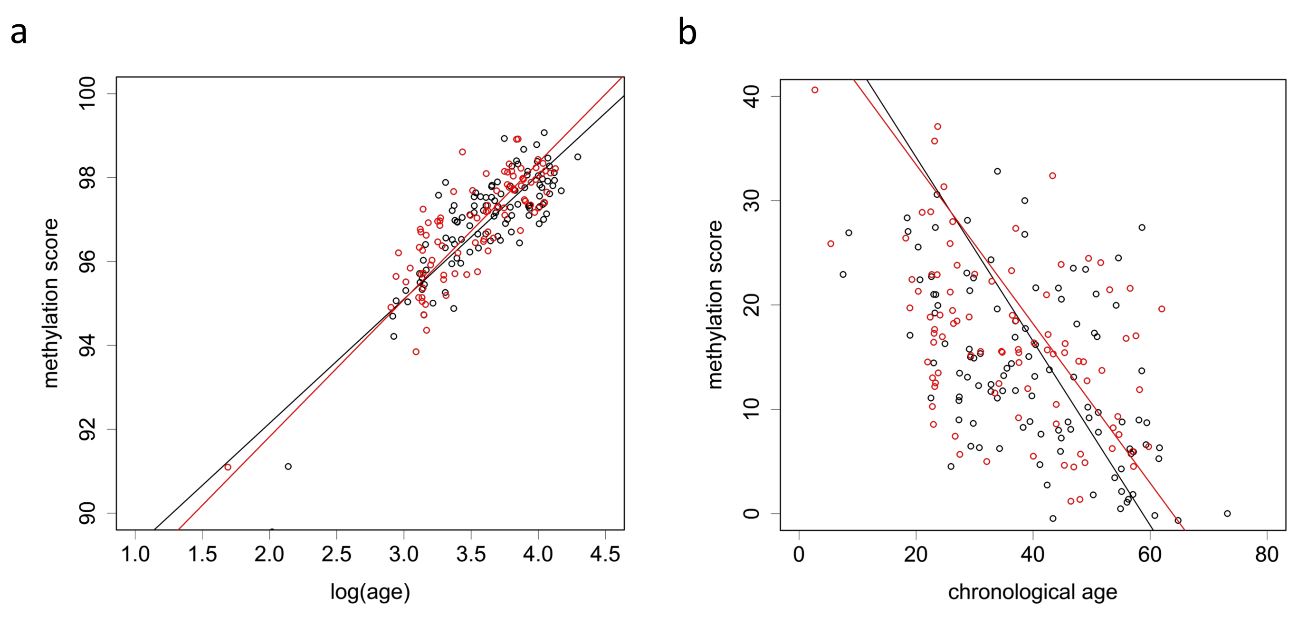


Supplementary Figure 2. (a) Distribution of logarithm of age and methylation score of ELOVL2 (black; male, red; female). (b) Distribution of chronological age and methylation score of EDARADD.


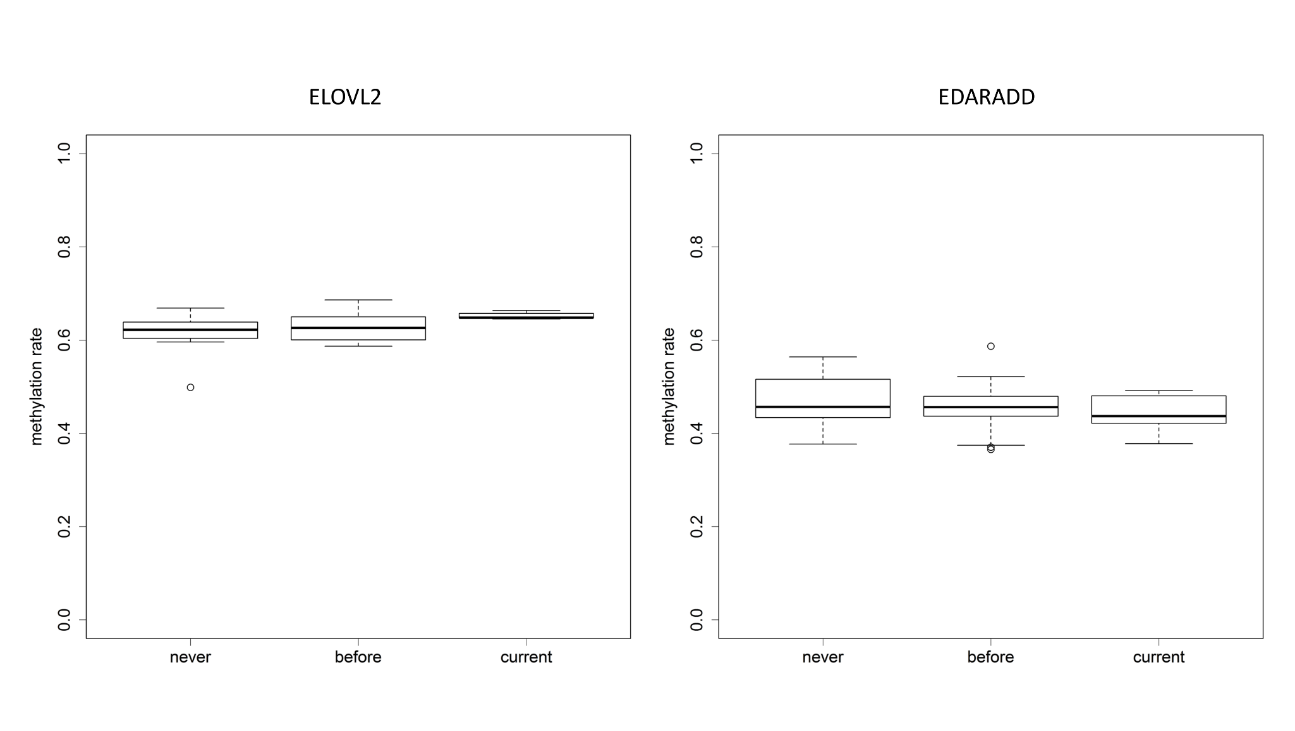


Supplementary Figure 3. Box plot of smoking habit and methylation rate at probe cg16867657 (ELOVL2) and cg09809672 (EDARADD). Methylation profile of 54 people (20 non-smokers, 29 ex-smokers, 5 current-smokers) in age from 49 to 51 years were retrieved from publicly available data set (GSE50660).


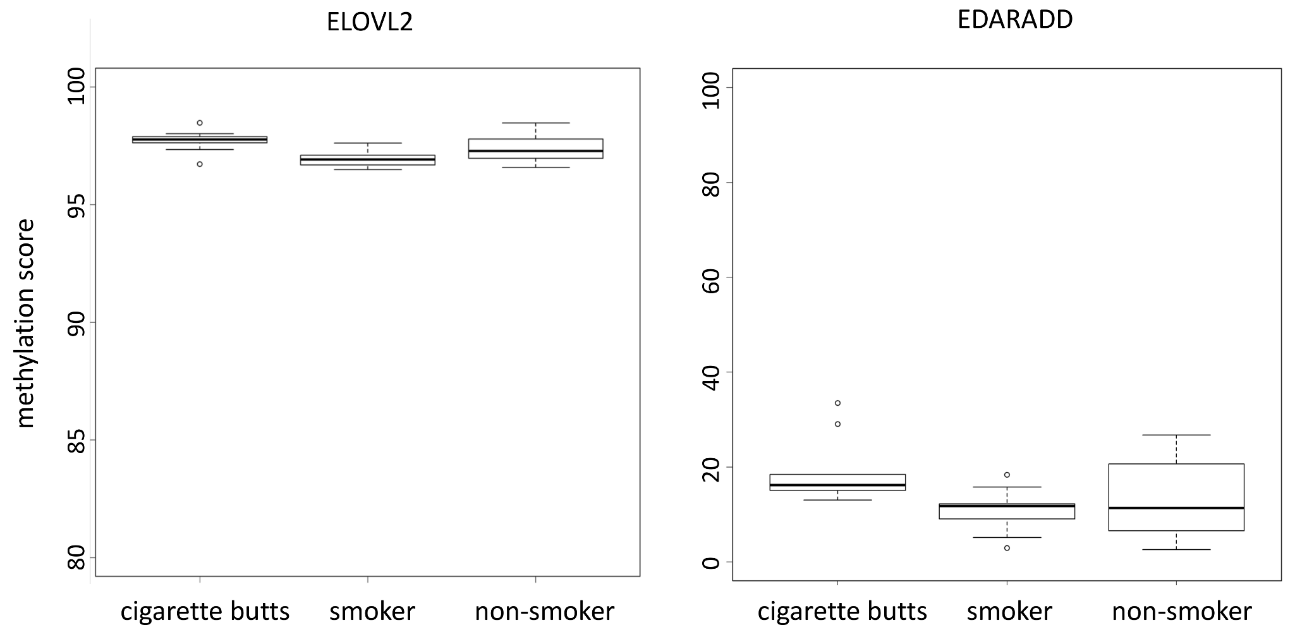


Supplementary Figure 4. Box plot of smoking habit and methylation score of ELOVL2 and EDARADD analyzed with MS-HRM. Cigarette butts and smokers’ saliva were collected from the same 9 people. Non-smokers’ saliva were collected from 7 people. All of the sample donors are aging 40 years.

Supplementary Table 1.

MAD in four age categories.

|  | Training set | Test set |
| --- | --- | --- |
| under 20 | 2.80 | 3.11 |
| 20-39 | 5.01 | 6.21 |
| 40-59 | 6.92 | 7.66 |
| over 60 | 11.8 |  |

Supplementary Table 2.

MAD differences between two analysing methods.

|  | samples | analysing method | MAD | |
| --- | --- | --- | --- | --- |
|  |  |  | training set | test set |
| our model　(*ELOVL2* only) | saliva | MS-HRM | 6.59 | 6.83 |
| model of Zbieć-Piekarska *et al.* | blood | pyrosequencing | 5.03 | 5.75 |
|  |  |  |  |  |

**Supplementary Reference**

1. Hamano, Y. *et al.* Forensic age prediction for dead or living samples by use of methylation-sensitive high resolution melting. *Leg. Med.* **21**, 5-10 (2016)
